# Colorectal cancer prevention for low-income, sociodemographically-diverse adults in public housing: baseline findings of a randomized controlled trial

**DOI:** 10.1186/1471-2458-9-353

**Published:** 2009-09-18

**Authors:** Lorna H McNeill, Molly Coeling, Elaine Puleo, Elizabeth Gonzalez Suarez, Gary G Bennett, Karen M Emmons

**Affiliations:** 1 Department of Health Disparities Research, University of Texas MD Anderson Cancer Center, Houston, USA; 2 Center for Community-Based Research, Dana-Farber Cancer Institute/Harvard School of Public Health, Boston, USA; 3 Division of Biostatistics and Epidemiology, University of Massachusetts, Amherst, USA; 4 Department of Psychology and Neuroscience, Duke University, Durham, USA

## Abstract

**Background:**

This paper presents the study design, intervention components, and baseline data from *Open Doors to Health*, a study designed to address social contextual factors in colorectal cancer (CRC) prevention for low-income, racial/ethnic minority populations.

**Methods:**

A cluster randomized design with 12 housing sites as the primary sampling units was used: 6 sites were assigned to a "Peer-led plus Screening Access" (PL) condition, and 6 were assigned to "Screening Access only" (SCR) condition. Study-related outcomes were CRC screening, physical activity (measured as mean steps/day), and multivitamin use.

**Results:**

At baseline (unweighted sample size = 1554), two-thirds self-reported that they were current with screening recommendations for CRC (corrected for medical records validation, prevalence was 52%), with half having received a colonoscopy (54%); 96% had health insurance. Mean steps per day was 5648 (se mean = 224), and on average 28% of the sample reported regular multivitamin use. Residents reported high levels of social support [mean = 4.40 (se = .03)] and moderately extensive social networks [mean = 2.66 (se = .02)].

**Conclusion:**

Few studies have conducted community-based studies in public housing communities; these data suggest areas for improvement and future opportunities for intervention development and dissemination. Findings from the randomized trial will determine the effectiveness of the intervention on our health-related outcomes as well as inform future avenues of research.

## Background

Colorectal cancer (CRC) is the 2^nd ^leading cause of cancer mortality in the US [1]. Over 49,000 deaths and over 100,000 new CRC cases are projected in 2008 [2]. While absolute CRC incidence and mortality rates have declined over the past 15 years, racial/ethnic minorities, and African Americans in particular, continue to have higher incidence and death rates compared to whites [2,3]. Recent evidence suggests that, in part because mortality rates among whites are dropping, the difference in CRC deaths between African Americans and whites has increased over the past 15 years, thus contributing to an increase in cancer-related health disparities [3]. Although rates for deaths attributable to CRC for Hispanic men and women have decreased or remained flat over this time period, similar to African Americans, they are more likely to be diagnosed with late-stage CRC and have lower survival rates compared to whites [4].

It is well-established that the majority of US cancer deaths, about 60%, could be prevented by improving lifestyle factors: reducing smoking and obesity, improving diet, increasing physical activity, and participating in screening for early cancer detection [3]. In particular, low rates of physical activity and participation in CRC screening significantly contribute to CRC incidence and death [5]. Nationally, less than 50% of adults meet physical activity recommendations of 30 minutes of moderate-intensity activity on 5 or more days per week [6]. Data from the 2006 Massachusetts BRFSS show that low-income adults (< $15,000/yr), African Americans (37%) and Latinos (44%) are least likely to meet these recommendations [7]. In addition to the aforementioned lifestyle factors, there is some evidence of a relationship between multivitamin use and chronic disease prevention. Studies show that on average, 52% of adults take multivitamins with lower intake among racial/ethnic minorities (26 - 36%) and those with less than a high school education (29-35%) [8,9]. Recent clinical reviews of this relationship are unclear as to the overall benefit of multivitamins in reducing risk for chronic disease, such as CRC, [10] but studies show it may be helpful [11,12].

It is estimated that screening alone can significantly reduce CRC incidence and mortality rates [13,14]. Yet, findings from national surveys show that almost half of all adults do not meet CRC screening guidelines [15]. For adults age 50 years and older, screening options include an annual fecal occult blood test (FOBT), sigmoidoscopy every 5 years, colonoscopy every 10 years, or double contrast barium enema every 5 years. Nationally, roughly 16% of adults have had a FOBT in the past year, with a greater majority receiving either an endoscopic screening test (i.e., sigmoidoscopy or colonoscopy) within the past 10 years (56%) [16]. Current CRC screening rates are highest for whites (62%) and lower among blacks (58%) and Latinos (46%); 57% of those insured are currently screened, compared to 25% among the uninsured. There are also large disparities in CRC screening rates based on educational attainment. In 2004, only 31% of those with less than high school education were currently screened, compared to 54% of college graduates [17]. In general, screening rates are increasing across all racial/ethnic groups; however, there remains a need to increase access to CRC screening for underserved populations [16].

**Figure 1:**
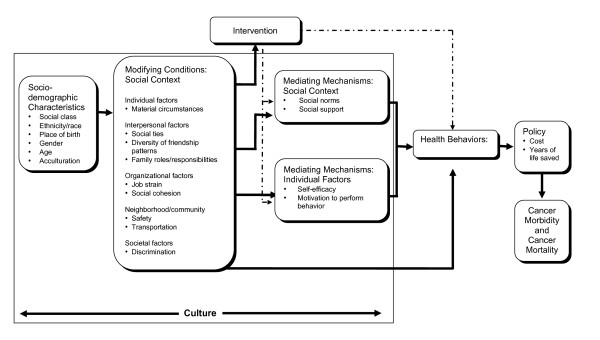
**Social contextual model**.

Racial/ethnic minorities and low-income populations report significant barriers to engaging in health promoting behaviors and getting screened for cancer. Sociodemographic characteristics and access to health care indeed determine patterns of engaging in cancer preventive behaviors. In addition to these long-established correlates of CRC prevention, social contextual factors are increasingly being examined as important influences on cancer prevention behaviors, particularly among underserved populations [18]. As part of our ongoing work to develop effective cancer prevention interventions for multi-ethnic, low-income populations, we have developed a conceptual framework for incorporating social context into the design and evaluation of interventions targeting risk behaviors (Figure 1). This model, based on the social ecological framework, draws on a range of social and behavioral theories to explicate the pathways by which social context may influence health behaviors. Social contextual factors cut across multiple levels of influence and include individual factors (e.g., material circumstances, psychosocial factors), interpersonal factors (i.e., social ties and family roles and responsibilities), organizational/systems factors (i.e., access to health care), and neighborhood and community factors (i.e., access to safe neighborhoods, transportation). These social contextual factors are shaped by a range of socio-demographic characteristics (i.e., social class, race and ethnicity) which taken together influence one's day-to-day realities and shape patterns of health behaviors and health outcomes.

Culturally appropriate, community-based interventions that focus on the day-to-day lives of socially and economically deprived populations are lacking. Public housing is increasingly being recognized as an important community-based setting for intervention delivery [19]. Public housing is comprised of low-income families, the elderly, those with disabilities, and large numbers of racial/ethnic minorities--populations that are at increased risk of poor health status. Currently there are 1.2 million households in public housing in the US [20]. Few interventions have specifically addressed the needs of those living in public housing. The few that have been conducted found that public housing residents are more likely to be obese, have chronic conditions, i.e., hypertension and diabetes, smoke, and be physically inactive [21]. However, low-income public housing environments also represent opportunities for public health improvement. It is a setting with great potential to affect the health of residents through positive mechanisms such as their peers, who are also their neighbors, and other social support networks that enable residents to share health-promoting information or easier access to health-related services such as food banks or health clinics [22]. Public housing residents are largely an insured group, given the income levels necessary to qualify for public housing [19,23], which may address a major barrier to screening, particularly in a state like Massachusetts where the state Medicaid program covers screening. Further, residents tend to live in these communities for a long period of time [24,25], thus making it also a relatively stable residential environment in which to conduct outreach.

*Open Doors to Health *is a theory-based peer-led intervention aimed at increasing CRC prevention behaviors in residents of low-income, ethnically diverse public housing communities. It sought to capitalize on social networks that exist within public housing and employed a peer-led, i.e., lay health advisor (LHA), model that used members of the target group for intervention delivery. Several behavioral studies have utilized peer-led models to increase the impact, reach, and sustainability of the interventions [26-29]. Typically in such peer-led interventions, members of the target population are selected, trained, and supported as they implement the intervention to promote changes in cancer prevention behaviors. This model has been employed and tested most extensively in low-income, ethnically diverse groups who carry a disproportionate share of the cancer burden. The majority of peer-led cancer prevention interventions have focused on increasing rates of cancer screening and/or target other cancer-related health behaviors. Paskett et al. [29] trained LHAs, individuals known and trusted in the community, to deliver health information to increase mammography utilization among a diverse sample of rural women. Compared to a control group, women in the intervention group were more likely to have received a mammogram at 12-month follow-up and reported reduced barriers for screening. Resnciow et al. [30] conducted a LHA intervention to increase fruit and vegetable intake among African American church goers and found a statistically significant increase in intake relative to controls at 6-month follow-up. These studies found that a peer-led model can be successfully implemented in racial/ethnic minority communities and result in significant behavior change over and above standard self-help interventions.

Relatively few studies have tested the peer-led model in interventions aimed specifically at CRC prevention, and those that have had limited impact on prevention behaviors. Campbell et al. [27] conducted an intervention trial testing the efficacy of using LHAs compared to the use of tailored print information for improving CRC screening, diet, and physical activity behaviors among African American church members. Findings showed that the LHA intervention arm failed to demonstrate effectiveness in any of the target health behaviors, likely due to limited reach of the LHAs to the target audience. However, church members who did talk with a LHA were twice as likely to obtain CRC screening compared to those who did not talk to a LHA. Few studies have evaluated the impact of the lay health advisor or peer leader model in the context of implementation within low-income housing, and to our knowledge none have addressed CRC screening in this setting.

The goal of the *Open Doors to Health *intervention was to build upon the strengths of using a social contextual framework to improve CRC risk factors in low-income public housing residents. Using a peer-led model, we sought to work through the already-existing social networks in the housing site to address individual and interpersonal factors, as well as neighborhood and organizational factors that make adopting healthful behaviors more challenging for racial/ethnic minorities and lower income populations. The peer-led model also fosters service delivery sustainability such that after the intervention period is over trained LHAs will retain the ability to provide advice and assistance to housing residents. The purpose of this paper is to describe the *Open Doors to Health *study design and intervention components, and to present the demographic characteristics of the study population by age (under 50 years old/age 50 and above) and in relation to our conceptual model. This randomized controlled trial was designed to increase CRC screening, physical activity, and multivitamin use among low-income public housing residents; a key aim is to determine ways in which attending to the social context in low-income, ethnically diverse populations may address disparities in CRC preventive behaviors.

## Methods

### Study Design

These data were collected as part of *Open Doors to Health (ODH)*, an NCI-funded study focused on CRC prevention delivered through low-income housing. This study used a cluster randomized design, with 12 housing sites as the primary sampling units. The housing site was the unit of randomization; six sites were assigned to a Peer-led plus Screening Access condition (PL), and six sites were assigned to Screening Access only (SCR) condition. In both the PL and SCR conditions, *ODH *offered equivalent access to screening through outreach and provision of information about all screening options including FOBT and endoscopy (i.e., colonoscopy/sigmoidoscopy), as well as expedited access to appointments at two local hospitals for those choosing endoscopy.

Secondary sampling units were individuals within the site. Unequal probability sampling across housing sites was employed due to the varying size of housing sites. In half of the sites (with population size less than 300 residents), the full population was sampled; in the remaining sites, with populations greater than 300, sampling was conducted to obtain an approximate 35% sample with a minimum of 250 subjects per site. Site participants were enrolled into the study in two housing units at a time. At the conclusion of the data collection period, the sites were randomly assigned to study condition. This strategy is commonly used in cluster randomized trials, such as those conducted within worksite and school settings [31]. It was our initial goal to match the housing sites in pairs based on resident demographics. However, there were no differences between intervention and control sites in these demographics at baseline.

### Setting

The housing sites in this study are comprised of both elderly and family sites. Ten were a mix of family and elderly, and two were senior housing sites. We worked through the management companies to introduce the study to the housing site authorities, either the Board or Tenants' Council, or the owners of the housing site. After initial meetings were conducted with the housing site leadership, the study was then introduced at a board, tenants' or community meeting. We subsequently completed a memorandum of understanding, which articulated the responsibilities and benefits of study participation, with each participating community. Of the 14 sites we approached, 12 showed interest in the research and agreed to participate.

### Sample

Participant recruitment began with housing site representatives sending letters announcing the study to their eligible residents, including the opportunity to opt out of contact. Eligibility criteria for the study survey included: (1) residence in the housing community; (2) age of at least 18 years; (3) English or Spanish fluency; and (4) not currently being treated for cancer. The study protocol was approved by the Human Subjects Committee at the Harvard School of Public Health.

An initial sample of 3688 residents was drawn, from which 747 (20%) were deemed ineligible, leaving an eligible sample of 2941 individuals. Of these, 828 (28%) refused participation, and 559 (19%) were never reached. Enrollment and baseline surveys were obtained on 1554 participants (53% response rate; range: 34% to 92% participation rate across the different housing sites). Participants provided informed consent and completed the interviewer-administered survey in either English or Spanish. Each person received $25 as monetary compensation for completing the baseline survey.

### Intervention Messages and Conditions

#### Intervention messages

The *ODH *study recommendations for CRC prevention were: (1) if you are 50 or over, you should get screened for CRC; (2) get 30 minutes of moderate to vigorous exercise on 5 days each week; and (3) take a multivitamin with folic acid every day. The intervention was delivered in both English and Spanish over 21 months in two housing sites at a time, and consisted of both message delivery and facilitation of access to CRC screening and to physical activity opportunities.

There were two intervention conditions: 1) *Peer-led plus Screening Access (PL): *The PL intervention condition was designed to increase access to screening, influence social norms and increase social support for behavior change, address social and environmental barriers to participation in CRC prevention, and bring sustainable resources for prevention to the housing sites through extensive involvement of peer leaders and the development of a resident working group. The intervention also attended to the social context by identifying physical features of the housing sites, through the implementation of an environmental audit, that were amenable to change and if changed could potentially increase physical activity among the residents; *2) Screening Access only (SCR): *In sites randomized to the SCR condition, materials containing the CRC prevention messages were mailed to housing site residents twice, once during each intervention year. These mailings included a letter encouraging people age 50 and over to talk to their doctors about CRC screening and to contact *ODH *staff if they would like help making a screening appointment. In addition, participants were offered access to CRC screening, equivalent to that offered in the PL intervention. Environmental audits were also conducted at baseline at SCR housing sites in order to document the physical environmental condition of each housing site; however, no interventions to make environmental changes for physical activity were conducted at these housing sites.

#### Peer-led plus Screening Access (PL) intervention condition

The PL intervention condition was supported by three key efforts: (1) Peer Leaders (PLs) (outreach, intervention implementation); (2) Resident Working Group (tailoring activities to the housing community, planning and implementation; reach); and (3) *ODH *staff/technical assistance. The PL intervention was implemented in close collaboration with housing site residents who were recruited and trained as *ODH *Peer Leaders (2-3 per housing site; 14 total). PLs participated in group training with all PLs from different housing sites, thus promoting idea sharing within the PL network and connectedness to the project as a whole. All PLs participated in twelve hours of initial training, with an additional 1.5 hours of training every other month. Topics included the CRC screening process, barriers, outreach, starting and leading walking clubs, event planning, and the *ODH *intervention messages. In addition, during the every other month sessions PLs built support, identified what was working and not working at their communities, and strategized about how to increase participation by residents to intervention activities. The PLs received a small stipend for their efforts.

In the first year of the study, nine focus groups in English and three in Spanish were conducted, as well as participant observations, and key informant interviews in all housing sites to include input from our intended audience in intervention design. The qualitative research focused on exploring: (1) community and social context, which included cultural, religious, language, neighbors, how invested people were in the housing site, neighborhood safety, relationship with management/board, and distrust of research; (2) health concerns; (3) physical activity, including identifying personal and environmental facilitators and barriers; and (4) on-going programs and activities at each community, including activity types, issues, attendance and incentives. This allowed us to understand what was important to each housing community and how to deliver the CRC prevention messages respecting the culture, language, and incorporating other health issues concerning to the community, like hypertension and diabetes, in our intervention messages and activities.

Each PL was expected to spend between two and five hours each week on intervention activities, including outreach to housing site residents, participation in monthly RWG meetings, regular contact with *ODH *staff, and co-leadership of intervention activities, described in more detail below. In addition to involvement in the implementation of all intervention activities, PLs participated in the Resident Working Group (RWG), a small group of resident volunteers that met monthly to provide feedback and input on the *ODH *peer-led intervention. *ODH *staff and PLs also worked closely with key leaders and groups at the housing sites, such as management companies and resident boards, to help ensure successful and sustainable intervention implementation. *ODH *also offered support for screening through individual counseling and provision of information, endoscopy appointments at two local hospitals within six weeks of referral by the patient's primary care provider, user-friendly screening preparation instructions, appointment reminders, and transportation to appointments. The PL intervention was designed to deliver CRC prevention messages working within the social context of the housing site residents, as well as to develop sustainable expertise within the housing sites.

Messages were delivered through the following activities, all of which were implemented at each housing site with the RWGs' input and staggered over the 21-month intervention period (see Table 1): (1) eight community events (i.e., "screening bingo," dance event, health fair); (2) nineteen discussion groups (delivered dialogue education re the outcomes of interest); (3) monthly poster campaigns featuring intervention messages and sometimes related photos and quotes from residents and PLs; (4) resource boards updated every three months; (5) ongoing outreach and follow-up via telephone and in-person contact delivered by the PLs and *ODH *staff; and (6) weekly on-site physical activity series and walking club for four 8-week periods. In each intervention year, the intervention dose included an 8-week walking club, as well as an 8-week non-walking group physical activity series. Each series facilitated moderate exercise for at least one hour per week and was held within or around the housing sites. Peer leaders were trained and supported in leading the walking clubs, including advertising, attendance taking, warm-up/stretching, safety, proper clothing/equipment, and group dynamics. In addition, some housing sites chose to expand and/or lengthen this menu of intervention activities.

**Table 1:** Intervention components and doses by year (Note that year = 10.5 months)

**Intervention component**	**Year 1 dose (per site)**	**Year 2 dose (per site)**
Events	Screening focus:	Screening focus:
	Bingo (1)	Bingo (2)
	Health fair (1)	Physical activity focus:
	Physical activity focus:	Walking or dance event (1)
	Walking event (1)	
	Dance event (1)	

Discussion groups	Screening focus: 6	Screening focus: 4
	Physical activity focus: 4	Physical activity focus: 4
	Screening & physical activity: 3	Screening & physical activity: 2

Poster campaigns	10.5 (1 per month)	10.5 (1 per month)

Resource boards	3 rounds	3 rounds

An environmental audit is a tool used to systematically use direct observation to characterize the social and physical housing site environment. It captures the presence, absence, and/or quality of resources that are thought to be important in promoting physical activity [32,33]. We used an audit tool originally developed for use in worksite settings (The Checklist of Health Promotion Environments at Worksites)[34], and modified it for use in a public housing setting. The audit was divided into two sections: 1) indoor environment, i.e., stairwells, signs and posters promoting physical activity; indoor physical activity equipment; and 2) outdoor environment, i.e., benches, playgrounds, sidewalks, open space that could be used for physical activity. For the purposes of the audit, we included in the outdoor environment only property owned by the housing site or resources that can visibly be seen from the housing site, such as parks.

Separately, two trained project staff walked through each intervention housing site and conducted the audit. Findings were developed into a report that summarized the findings and highlighted areas for potential improvement for the PL intervention condition only. For example, if audit findings revealed that stairwells were locked at all times and thereby limited the stairwells as a vehicle for physical activity among the residents, the report would identify this as a potential area for improvement. This report was presented to each housing site in a meeting of key decision makers and advocates at the sites, including Peer Leaders, Resident Working Group members, and housing site management. At this meeting, each housing site was encouraged to ask questions about the findings and choose at least one of the areas for improvement. *ODH *staff then worked with each housing site on the selected task and identified opportunities for effecting change, such as working with the housing site management, the City of Boston, or other agencies to improve the housing site environment for physical activity.

### Measures

#### Sociodemographic characteristics

Sociodemographic variables collected in the interviewer administered baseline survey include sex, age, marital status (married/not married) race/ethnicity (categorized as black, white, Hispanic, and other), highest level of education completed, and employment status (working full-time, working part-time, disabled, not working). We also assessed yearly household income, poverty status and financial situation. Yearly household income (six response options ranging from less than US $10,000 to at least US $50,000) and the number of people supported by this income were used to measure poverty status (dichotomized as being above or below the poverty level based on the 2005 federal poverty guidelines on income and household size)[35]. Participants were also asked about their perception of the financial status of their household (comfortable with some extras, enough but no extras, have to cut back, or cannot make ends meet). We assessed immigrant status by asking participants their birthplace and their first or native language. Lastly, we also assessed whether participants had health insurance (public insurance, private insurance, combination of public and private insurance, or no insurance).

#### Social contextual factors

Social contextual factors were measured at the individual, interpersonal and societal levels. At the individual level, participants were asked about their use of media sources. Participants were asked to report the number of hours per weekday and weekend day they watch **television **[36] and whether they owned a home **computer. Mental health status **was assessed by asking participants whether they experienced depressed mood (how often they were downhearted and sad) in the last 4 weeks. Overall **health status **was assessed by asking participants whether their physical health restricted moderate exercise in the last 4 weeks.

At the interpersonal level, marital status, number of close friends, number of close family members, and active membership in organizations (religious, professional, community, civic, etc) were combined to form a continuous measure of the number of **social network ties **ranging from 0 to 4, with a higher score indicating a greater social network [37]. **Social support **was assessed by asking participants about emotional support from family and friends, support when sick, help with household tasks, financial support, and help getting to the doctor. A single social support variable was created by adding the number of responses to the five questions that indicated at least some support, with a range of 0 to 5 [38]. Higher scores indicated greater social support. Participants were asked about their various **family role responsibilities**, which included "earning money to support the family," "taking care of children," and "taking care of another household." The measure of multiple roles was computed as the number of family roles for which the participant was mostly or fully responsible (0 to 3). To determine **role conflicts**, participants were asked whether their daily activities made conflicting demands on them. **Social norms **for physical activity and CRC screening were assessed by asking participants how many of their family and friends get at least 30 minutes of exercise per day and have ever had a CRC screening test. Response options were few or none, some, most or all [39].

At the societal level, participants were asked about neighborhood safety, racial/ethnic discrimination, perception of neighborhood resources and neighborhood social cohesion. **Neighborhood safety **was assessed by asking whether participants felt safe walking alone in their neighborhood during the daytime and at night [40]. Participants were also asked how often they ever felt discriminated against based on their race/ethnicity in 5 situations: 1) getting a job; 2) at work; 3) getting housing; 4) getting medical care; and 5) in the street or in a public setting. For each of the five items, participants chose from the following response options: *Never*; *Once*; *2-3 times*; or *4 or more times*. The variable was further dichotomized 'ever' vs. 'never' experienced discrimination [41].

Perceptions of **neighborhood resources **were assessed by asking participants whether there were many places to go to within easy walking distance of their home. Finally, to assess **social cohesion **in the housing community, we asked respondents to report their agreement with five statements: (1) people around here are willing to help their neighbors; (2) this is a close-knit neighborhood; (3) people in this neighborhood can be trusted; (4) people in this neighborhood generally do not get along with each other; and (5) people in this neighborhood do not share the same values. Item responses were reversed for the first three statements and then responses to the five items were averaged. The summary score ranged from 1 to 4, with a higher score indicating higher social cohesion [42].

#### Primary outcomes

**Physical activity **was objectively measured using pedometers and captured total steps per day for three days. Our pedometer sampling protocol is described elsewhere in greater detail [43]. All study participants were enrolled in the physical activity protocol unless deemed ineligible; 1185 had complete pedometer data. The study pedometers (Yamax SW200, Lee's Summit, Mo) demonstrate high concordance with accelerometers (motion sensors that use a piezoelectric transducer to objectively measure physical activity intensity and duration with high precision) under both laboratory conditions and in field settings [44]. Participants wore the pedometer from the time they woke in the morning until they went to bed. Mean steps per day are presented. **Multivitamin use **was assessed by asking participants the number of days per week they take multivitamins [45,46]. Regular multivitamin use was characterized as use on 6 or more days. **Colorectal cancer screening **was assessed using questions developed and adopted by NCI as gold standard self-report measures of screening [47]. Following guidelines from the ACS and US Preventive Services Task Force, participants were considered current with CRC screening if they reported having a FOBT within one year of the survey, a sigmoidoscopy within five years, and/or a colonoscopy within ten years. Participants who reported more than one screening test were credited with having the most efficacious screening method, with ranking as follows: 1) colonoscopy, 2) sigmoidoscopy, and 3) FOBT.

### Data Analysis Plan

For all analyses, based on the cluster design, data are weighted up to the population size within each housing site (with a total weighted size of 2271). Following the methods presented in Korn and Graubard [48], we first calculated base weights which weighted the sample to reflect the population size within each housing site. Our nonresponse adjustment to these weights were based on gender and age category (< 50 years, ≥ 50 years) because we assumed that these variables defined response rate cells in which individuals had equal probability of responding. Appropriate analyses were conducted using SUDAAN and SAS statistical software for clustered data. Frequency distributions (for categorical variables) and estimates of means and standard deviations (for continuous variables) were assessed for distributional assumptions and outliers.

## Results

### Demographics

Demographic characteristics are presented in Table 2. All percentages reported in this table are weighted to reflect population percents. The majority of the participants were racial/ethnic minorities (95%), women (74%), not married (68%), were not working or disabled (64%), and had a high school education or less (65%). Slightly more than half of the sample (54%) was 50 years of age or older; the mean age was 51 years (se = 0.4).

**Table 2:** Demographic and health behavior characteristics of the study population

**Demographics** **Characteristics**	**Unweighted** **sample size** **(N = 1554)**	% *
Sex		
Male	421	26%
Female	1133	74%

Race/ethnicity		
Black	793	48%
White	71	5%
Hispanic	630	44%
Other	50	3%

Marital status		
Married	376	25%
Not married	1063	68%

Education		
Less than high school	617	39%
High school or equivalent	427	26%
Greater than high school	505	32%

Employment status		
Working full-time	330	22%
Working part-time	206	14%
Disabled	317	23%
Not working	600	41%

Yearly household income		
≤ $10,000	541	43%
$10,000-19,999	391	31%
≥ $20,000	337	26%

Poverty status		
Above poverty level	724	51%
Below poverty level	684	49%

Financial situation		
Comfortable with some extras	361	23%
Enough but no extras	461	29%
Have to cut back	377	26%
Cannot make ends meet	211	13%

English native language		
Yes	866	47%
No	686	52%

Immigrant		
Yes	700	48%
No	852	52%

Regular health care provider		
Yes	1343	87%
No	203	13%

Health insurance		
None	65	4%
Public Insurance Only	1012	66%
Private insurance only	388	25%
Public+ Private	80	5%

Current with CRC screening (≥ 50 only)		
Yes	563	67%
No	273	33%

CRC test (≥ 50 only)		
FOBT	215	24%
Sigmoidoscopy	125	14%
Colonoscopy	453	54%

Intention to get screened (< 50 only)		
Yes	411	66%
No	211	34%

Takes multivitamins (days/week)		
< 6	1105	72%
≥ 6	449	28%

Age; mean (se)	50.7 (0.4)	

Steps/day; mean (se)	5648 (22.0)	

Body mass index (se)	30 (0.3)	

*Percents are based on weighted values except where noted

Although 74% of the participants had household incomes of less than $20,000 per year, only about half (49%) were below the poverty level as defined by federal poverty guidelines. Almost half of all participants (48%) were not born in the US, and 52% percent reported English as a second language. The majority reported having access to a regular health care provider (87%) and having health insurance (96%). Overall, the mean body mass index of the sample was 30.0 (se = 0.3).

### Social contextual factors

Table 3 displays the prevalence of social contextual factors in this population. Overall, almost half (48%) of all participants reported experiencing some form of discrimination at least once. The most frequently reported instances of discrimination were at work (29%) and on the street or in a public setting (25%) (data not shown). Participants also reported living in a moderately cohesive neighborhood [mean = 2.66 (se = .02)]

**Table 3:** Social contextual characteristics of the study population

**Social Contextual** **Characteristics**	**Unweighted sample size** **(N = 1554)**	**%**
Racial/ethnic discrimination		
Ever	520	48%
Never	535	52%

Perceived daytime safety		
Safe	1118	80%
A little unsafe	228	16%
Unsafe	57	4%

Perceived nighttime safety		
Safe	478	37%
A little unsafe	451	33%
Unsafe	423	30%

Social norms for CRC screening		
Most or all	117	8%
Some	227	15%
Few or none	587	37%
No answer/DK	623	40%

Social norms for physical activity		
Most or all	233	15%
Some	436	29%
Few or none	546	35%
No answer/DK	339	21%

Role conflict		
Yes	517	33%
No	885	57%
No answer/DK	152	10%

Neighborhood resources		
Strongly agree/somewhat agree	1302	84%
Strongly disagree/somewhat disagree	98	6%
No answer/DK	154	10%

TV use (hours/day)		
0	128	8%
> 0 to 2	402	26%
> 2 to 4	516	34%
> 4 to 6	264	17%
> 6	240	14%

Computer ownership		
Yes	724	48%
No	715	45%

Mental health status (downhearted and blue)		
A little, some, most or all of the time	925	63%
Rarely or not at all	536	37%

Health status (physical health restricts exercise)		
Yes	692	45%
No	861	55%

	Mean (SE)	

Social ties/networks (0-4)	2.66 (0.02)	

Social support (0-5)	4.40 (0.03)	

Role responsibilities (0-3)	1.41 (0.02)	

Social cohesion (1-4)	2.49 (0.02)	

*Percents are based on weighted values except where noted

Interpersonally, most residents reported high levels of social support [mean = 4.40 (se = .03)] and moderately extensive social networks [mean = 2.66 (se = .02). Thirty-three percent reported some difficulty managing their multiple roles and noted some responsibility for being the primary caretaker and money earner in the household. Low social norms for physical activity and CRC screening were reported, with 44% and 23% reporting that some/most or all of their family and friends engage in physical activity or get screened for cancer, respectively. A large majority of residents felt safe in their neighborhoods during the day (80%), but only 37% perceived their neighborhood to be safe at night.

There was great variability in television viewing among the participants; 31% watching 4 or more hours per day and 34% watching less than 2 hours per day. Computer ownership was almost evenly split, with 48% of residents owning a home computer. Sixty-three percent of the participants reporting feeling downhearted and blue some, most or all of the time in the past 4 weeks, and 45% reporting that their physical health restricted exercise in the past 4 weeks.

### Physical activity

Table 4 presents prevalence of health behaviors by age (< 50 years and ≥ 50 years). Mean steps per day for the sample was 5648 (se mean = 224), typical of daily activity without exercise, i.e., low active [49]. Overall, those under 50 years old were more active, registering an average 6647 steps/day (se mean = 301). The 50 and over age group registered an average of 4659 steps/day (se mean = 184). Regardless of age, men were more active than women, whites were more active than non-whites; also more active were those who were employed, those with greater incomes, those without health insurance, and those whose native language was something other than English or Spanish. Least active among all groups were blacks over 50 years old (3968 steps/day) and unemployed adults over 50 years old (3691 steps/day).

**Table 4:** Prevalence of health behaviors by demographic characteristics, under and over age 50

**Demographics** **Characteristics**	**< 50 y.a.** **(N = 718)**			**50 y.a.** **(N = 836)**	
	**Physical activity steps** **mean (se)**	**Multivitamins** **(6 +Days/Week)**	**Screened**	**Physical activity steps** **mean (se)**	**Multivitamins** **(6 +Days/Week)**

Overall	6647 (301)	17%	66%	4659 (184)	37%

Sex					
Male	8395 (388)	16%	68%	5721 (289)	33%
Female	6129 (165)	18%	66%	4188 (155)	39%

Race/Ethnicity					
Black	6560 (218)	18%	68%	3968 (188)	41%
White	8479 (651)	24%	63%	5481 (490)	45%
Hispanic	6537 (250)	16%	65%	5202 (238)	31%
Other	7533 (934)	21%	76%	5186 (859)	47%

Education					
Less than high school	6270 (408)	18%	70%	4651 (200)	35%
High school or equivalent	6733 (276)	14%	62%	4447 (309)	37%
Greater than high school	6755 (214)	19%	64%	4865 (276)	43%

Employment Status					
Working full-time	7768 (299)	16%	64%	7209 (480)	30%
Working part-time	7322 (360)	16%	68%	5680 (461)	49%
Disabled	4041 (269)	22%	72%	4710 (292)	34%
Not working	5761 (228)	18%	68%	3691 (161)	38%

Annual Household Income					
≤ $10,000	6134 (266)	18%	69%	4595 (222)	36%
$10,000-19,999	6361 (319)	17%	70%	4680 (310)	42%
≥ $20,000	7066 (276)	20%	66%	5409 (336)	37%

Native Language					
English	6648 (159)	18%	69%	4128 (184)	43%
Spanish	6532 (265)	17%	66%	5136 (238)	31%
Other	7393 (628)	16%	55%	5579 (529)	34%

Health Insurance					
None	7612 (908)	9%	55%	5953 (687)	27%
Public insurance only	6124 (207)	16%	67%	4413 (166)	36%
Private insurance only	7323 (269)	22%	64%	5603 (333)	39%
Public+ private	6159 (520)	17%	75%	3709 (617)	46%

Age; mean (se)		38 (0.7)	65 (0.3)		66 (0.5)

### Multivitamin use

Overall, 28% of the sample reported regular multivitamin use (defined as 6 or more days per week)[9]. Large age differences were noted; 17% of those under 50 years old vs. 37% of those 50 years old and over reported regular multivitamin use. In the under 50 age group, whites, those disabled, and those with private insurance reported greater multivitamin use. Among those over 50, use was highest among women (39%), those with high education attainment (43%), those who had public+private health insurance (46%), those who worked part-time (49%), and those who reported English as their first language (43%). Regardless of age, multivitamin use was lowest among Hispanics and those without health insurance.

### Colorectal cancer screening

We assessed self-reported CRC screening in the 50 and over age group only. Current screening was reported for 67% of the sample (corrected for validation, prevalence was 52%)[50]. Men and women were equally as likely to be screened (68% vs. 66%, respectively). Whites (63%) and those without health insurance (55%) had the lowest reported screening rates whereas participants with less than high school education (70%), those with public+private health insurance (75%), and disabled adults (72%) had the highest screening rates.

## Discussion

Cancer-related health disparities of incidence, morbidity, and mortality differentially affect racial/ethnic minorities and lower income groups. Recently, there has been an increased awareness of the relationship between poorer health outcomes in these groups and the social, economic, political, and cultural environments in which they live. This is particularly true for persons living in low-income public housing, environments which are often located in distressed neighborhoods with few health promoting resources. Public housing residents report poor health status, chronic disease conditions, and poor health behaviors [23,51]. Thus it is imperative that health promotion interventions targeted and tailored to racial/ethnic minorities and low-income groups consider this constellation of risk factors in order to be effective at closing the health disparities gap. Baseline findings from *Open Doors to Health (ODH) *suggests that segments of this population, i.e., Hispanics and unemployed adults, are at even greater increased risk for CRC given their low socioeconomic position and low levels of physical activity.

Data from the 2006 Behavioral Risk Factor Surveillance System show that on average 91% of Massachusetts adults had health insurance--this figure is substantially lower for blacks (81%), Hispanics (76%), and those with low incomes (86%) [7]. In contrast to national estimates, the majority of *ODH *study participants had health insurance coverage (96%), either through public, private, or a public+private; this population most likely qualified for health care based on income and/or age requirements. This may in part explain the high rate of CRC screening in this population, in addition to CRC testing as a covered Medicare benefit nationally and as a covered Medicaid benefit in Massachusetts [15]. Comprehensive health insurance is indeed a benefit for those seeking to obtain CRC screening given the often high co-payment associated with some of the screening tests. Nationally, about 60% of adults are currently screened for CRC [16]; among our participants 66% of those age-eligible for CRC were up-to-date on their screening [50]. These findings indicate a strong relationship between access to care and screening uptake; however with almost universal health coverage, 34% of those over 50 years old were not CRC current. This indicates an opportunity for improvement and a need to better understand the mechanisms by which social contextual factors, such as the cost of co-payments, that might hinder participation in screening among those with insurance. Health insurance makes obtaining screening more financially feasible, and facilitates access to other health promoting services. However, it can be argued that certain behaviors, such as physical activity or multivitamin use are heavily influenced by individual and broader social contextual factors, such as perceived benefit, cost, time, and access to resources.

Overall, daily, regular multivitamin use was very low in this population (28%) and even lower among those under 50 years old (17%); however, use among Hispanics regardless of age (16% for Hispanics under 50 years; 31% for Hispanics age 50 and up), was much lower than all other racial/ethnic groups. This finding highlights an opportunity for intervention. Understanding and resolving barriers to multivitamin use among Hispanics may help to reduce CRC risk in this group. A 2001 study found that multivitamins use is predictive of CRC screening among women--women who took a daily supplement were 1.74-2.12 times more likely to be current with their CRC screening [52]. The inexpensive, simple act of taking a multivitamin could potentially be a cue to engaging in other health promoting behaviors. Our prior research studies focused on increasing multivitamin use among racial/ethnic minorities and lower SES groups found an almost 30% increase in multivitamin use at follow-up [45]; this increase could result in a significant (> 25%) reduction in CRC incidence as well as be an important facilitator for the reduction in other conditions such as cardiovascular disease, osteoporosis, and birth defects [53,54].

For good health, current physical activity recommendations are for adults to engage in at least 30 minutes of moderate-intensity physical activity on at least 5 days per week--the equivalent is the accumulation of 10,000 steps per day [49]. On average, study participants had levels of activity (~5600 steps/day), far lower than recommended levels [43]. In comparing racial/ethnic groups, blacks and Hispanics were the least active, and physical activity was lowest among blacks over 50 years old (mean = 3968 steps/day), indicative of a sedentary lifestyle and increased risk for chronic health conditions and poor health outcomes. Unemployed older adults had the lowest physical activity levels overall (3691 steps/day), even lower than those disabled (4041-4710 steps/day). Studies have shown a similar relationship between unemployment and physical inactivity, possibly due to the absence of job-related or transportation-related activity [55]. They may also be more likely to have other health conditions, i.e., obesity, and comorbities that increase sedentary behaviors [56]. Fewer studies focus on these populations (i.e., disabled, unemployed) in an effort to increase their activity levels.

In *Open Doors to Health*, we were focusing on salient social contextual factors that may have an impact on intervention effectiveness and health behavior change, such as interpersonal and neighborhood factors. Although the majority of participants felt that their neighborhoods were safe in the daytime (85%) and agreed there were many places to go within walking distance of their home (84%), the overall mean steps per day was low. Although unable to be determined from baseline findings, it is possible that many participants have free time in the evening and would choose to engage in physical activity at that time, but feel their neighborhood is unsafe at night (63%) [57]. Participants also reported high social support from family and friends, but moderate social networks, suggesting that although the breadth of their networks may be somewhat limited, they do perceive substantial support from their social networks. This has important implications for conducting health behavior change interventions in this group. For example, women of color are more likely to engage in physical activity with others than alone, and express need for social support to be active [58,59]. In *ODH*, we worked through their social networks and focused on social support to increase intervention reach and effectiveness. We sought to positively modify social norms for screening and physical activity, which are both currently very low, through on-site activities delivered and based in their community.

Targeting and working within the cohesive social structure of public housing sites in combination with efforts to improve the housing site environment represents an assets-oriented approach to improving health behaviors of public housing residents. Similar to interventions that have been effective in improving the health of worksite employees, [18,46] in this intervention, we sought to build upon the resources and assets, i.e., residents and management, already available in the housing sites. In fact, resident service coordinators at many of the housing sites already helped residents obtain health insurance and make medical appointments prior to the intervention, likely contributing to the high rates of CRC screening in this sample. Moreover, this also represents a potentially sustainable intervention as CRC prevention information and resources are retained at the level of the housing site and can represent a long-term strategy for improving the health behaviors of racial/ethnic minority and low-income groups with health insurance.

Several study limitations and strengths should be addressed. We achieved a response rate of 53% which ranged from a low of 34% to a high of 92% across the housing sites. Nevertheless, we targeted, recruited and enrolled 1554 participants, which represents a large, ethnically-diverse underserved population. This study has limited generalizability to other populations other than low-income, urban, racial/ethnic minorities living in public housing. In an effort to accurately and objectively report CRC screening rates in this population, we also validated self-reports of CRC screening with medical record verification in a sample subgroup.

## Conclusion

*Open Doors to Health *was designed to better understand and address the needs of low-income minorities in public housing to incorporate the complexities of their lives into a socially-and culturally-appropriate CRC prevention intervention. Intervention activities were specifically designed to target modifiable factors, such as social networks, neighborhood social cohesion, and access to health promoting resources (i.e., developing walking clubs) in an effort to strengthen both individual and organizational capacity for reducing cancer risk factors [18]. Few studies have conducted community-based studies in public housing communities; these data suggest areas for improvement and future opportunities for intervention development and dissemination. In particular, the high rate of participation in some CRC prevention behaviors (screening), and low participation in others (e.g. physical activity), suggests the importance of both health insurance coverage and access to preventive health programs, as all risk factors are not likely to be addressed by insurance alone. Findings from the randomized trial will determine the effectiveness of the intervention on our health-related outcomes as well as inform future avenues of research.

## Competing interests

The authors declare that they have no competing interests.

## Authors' contributions

LHM drafted the manuscript and led the environmental audit condition. EGS was the trial coordinator. MC was the peer-leader coordinator and drafted parts of the manuscript. EP performed the statistical analysis and drafted the results section. GGB led the physical activity pedometer protocol and helped draft the manuscript. KME was the principal investigator, conceived of the study, and participated in its design and coordination and helped to draft the manuscript. All authors read and approved the final manuscript.

## Pre-publication history

The pre-publication history for this paper can be accessed here:



## References

[B1] Jemal A, Siegel R, Ward E, Hao Y, Xu J, Murray T, Thun MJ (2008). Cancer statistics, 2008. CA Cancer J Clin.

[B2] American Cancer Society (2008). Cancer Facts and Figures 2008.

[B3] American Cancer Society (2007). Cancer Facts and Figures for African American 2007.

[B4] American Cancer Society (2006). Cancer Facts and Figures for Hispanics 2006-2008.

[B5] Kushi LH, Byers T, Doyle C, Bandera EV, McCullough M, McTiernan A, Gansler T, Andrews KS, Thun MJ (2006). American Cancer Society Guidelines on Nutrition and Physical Activity for cancer prevention: reducing the risk of cancer with healthy food choices and physical activity. CA Cancer J Clin.

[B6] Centers for Disease Control and Prevention (2007). Prevalence of regular physical activity among adults--United States, 2001 and 2005. MMWR Morb Mortal Wkly Rep.

[B7] Centers for Disease Control and Prevention (CDC) (2006). Behavioral Risk Factor Surveillance System Survey Data.

[B8] Radimer K, Bindewald B, Hughes J, Ervin B, Swanson C, Picciano MF (2004). Dietary supplement use by US adults: data from the National Health and Nutrition Examination Survey, 1999-2000. Am J Epidemiol.

[B9] Shelton RC, Puleo E, Syngal S, Emmons KM (2009). Multivitamin use among multi-ethnic, low-income adults. Cancer Causes Control.

[B10] Huang HY, Caballero B, Chang S, Alberg AJ, Semba RD, Schneyer CR, Wilson RF, Cheng TY, Vassy J, Prokopowicz G, Barnes GJ, Bass EB (2006). The efficacy and safety of multivitamin and mineral supplement use to prevent cancer and chronic disease in adults: a systematic review for a National Institutes of Health state-of-the-science conference. Ann Intern Med.

[B11] Giovannucci E, Stampfer MJ, Colditz GA, Hunter DJ, Fuchs C, Rosner BA, Speizer FE, Willett WC (1998). Multivitamin use, folate, and colon cancer in women in the Nurses' Health Study. Ann Intern Med.

[B12] Jacobs EJ, Connell CJ, Chao A, McCullough ML, Rodriguez C, Thun MJ, Calle EE (2003). Multivitamin use and colorectal cancer incidence in a US cohort: does timing matter?. Am J Epidemiol.

[B13] Selby JV, Friedman GD, Quesenberry CP, Weiss NS (1992). A case-control study of screening sigmoidoscopy and mortality from colorectal cancer. N Engl J Med.

[B14] Mandel JS, Church TR, Bond JH, Ederer F, Geisser MS, Mongin SJ, Snover DC, Schuman LM (2000). The effect of fecal occult-blood screening on the incidence of colorectal cancer. N Engl J Med.

[B15] Meissner HI, Breen N, Klabunde CN, Vernon SW (2006). Patterns of colorectal cancer screening uptake among men and women in the United States. Cancer Epidemiol Biomarkers Prev.

[B16] Smith RA, Cokkinides V, Brawley OW (2008). Cancer screening in the United States, 2008: a review of current american cancer society guidelines and cancer screening issues. CA Cancer J Clin.

[B17] Seeff LC, Nadel MR, Klabunde CN, Thompson T, Shapiro JA, Vernon SW, Coates RJ (2004). Patterns and predictors of colorectal cancer test use in the adult U.S. population. Cancer.

[B18] Sorensen G, Emmons K, Hunt MK, Barbeau E, Goldman R, Peterson K, Kuntz K, Stoddard A, Berkman L (2003). Model for incorporating social context in health behavior interventions: applications for cancer prevention for working-class, multiethnic populations. Prev Med.

[B19] Ahluwalia JS, Nollen N, Kaur H, James AS, Mayo MS, Resnicow K (2007). Pathway to health: cluster-randomized trial to increase fruit and vegetable consumption among smokers in public housing. Health Psychol.

[B20] U.S. Department of Housing and Urban Development (2007). HUD's Public Housing Program Homes & Communities.

[B21] Heinrich KM, Lee RE, Regan GR, Reese-Smith JY, Howard HH, Haddock CK, Poston WS, Ahluwalia JS (2008). How does the built environment relate to body mass index and obesity prevalence among public housing residents?. Am J Health Promot.

[B22] Fertig AR, Reingold DA (2007). Public housing, health and health behaviors: is there a connection?. J Policy Anal Manage.

[B23] Digenis-Bury EC, Brooks DR, Chen L, Ostrem M, Horsburgh CR (2008). Use of a population-based survey to describe the health of Boston public housing residents. Am J Public Health.

[B24] Newman SJ, Harkness JM (2002). The long-term effects of public housing on self-sufficiency. J Policy Anal Manage.

[B25] Shankar S, Klassen AC, Garrett-Mayer E, Houts PS, Wang T, McCarthy M, Cain R, Zhang L (2007). Evaluation of a nutrition education intervention for women residents of Washington, DC, public housing communities. Health Educ Res.

[B26] Earp JA, Eng E, O'Malley MS, Altpeter M, Rauscher G, Mayne L, Mathews HF, Lynch KS, Qaqish B (2002). Increasing use of mammography among older, rural African American women: results from a community trial. Am J Public Health.

[B27] Campbell MK, James A, Hudson MA, Carr C, Jackson E, Oakes V, Demissie S, Farrell D, Tessaro I (2004). Improving multiple behaviors for colorectal cancer prevention among African American church members. Health Psychol.

[B28] Katz ML, Tatum CM, Degraffinreid CR, Dickinson S, Paskett ED (2007). Do cervical cancer screening rates increase in association with an intervention designed to increase mammography usage?. J Womens Health (Larchmt).

[B29] Paskett E, Tatum C, Rushing J, Michielutte R, Bell R, Long Foley K, Bittoni M, Dickinson SL, McAlearney AS, Reeves K (2006). Randomized trial of an intervention to improve mammography utilization among a triracial rural population of women. J Natl Cancer Inst.

[B30] Resnicow K, Campbell MK, Carr C, McCarty F, Wang T, Periasamy S, Rahotep S, Doyle C, Williams A, Stables G (2004). Body and soul. A dietary intervention conducted through African-American churches. Am J Prev Med.

[B31] Murray DM (1998). Design and analysis of group randomized trials.

[B32] Pikora TJ, Bull, Fiona CL, Jamrozik, Konrad, Knuiman, Matthew, Giles-Corti, Billie, Donovan, Rob J (2002). Developing a reliable audit instrument to measure the physical environment for physical activity. Am J Prev Med.

[B33] Hoehner CM, Ivy A, Ramirez LB, Meriwether B, Brownson RC (2006). How reliably do community members audit the neighborhood environment for its support of physical activity? Implications for participatory research. J Public Health Manag Pract.

[B34] Oldenburg B, Sallis JF, Harris D, Owen N (2002). Checklist of Health Promotion Environments at Worksites (CHEW): development and measurement characteristics. Am J Health Promot.

[B35] United States Department of Health and Human Services (2005). 2005 Federal Poverty Guidelines.

[B36] Nelson DE, Kreps GL, Hesse BW, Croyle RT, Willis G, Arora NK, Rimer BK, Viswanath KV, Weinstein N, Alden S (2004). The Health Information National Trends Survey (HINTS): development, design, and dissemination. J Health Commun.

[B37] Barrera M, Sandler IN, Ramsey TB (1981). Preliminary development of a scale of social support: studies on college students. Am J Community Psychol.

[B38] Berkman LF, Syme SL (1979). Social networks, host resistance, and mortality: a nine-year follow-up study of Alameda County residents. Am J Epidemiol.

[B39] Raven B, Rubin J (1976). Social psychology: people in groups.

[B40] Troutt DD (1993). The thin red line: how the poor still pay more.

[B41] Krieger N, Smith K, Naishadham D, Hartman C, Barbeau EM (2005). Experiences of discrimination: validity and reliability of a self-report measure for population health research on racism and health. Soc Sci Med.

[B42] Sampson RJ, Raudenbush SW, Earls F (1997). Neighborhoods and violent crime: a multilevel study of collective efficacy. Science.

[B43] Bennett GG, Wolin KY, Puleo E, Emmons KM (2006). Pedometer-determined physical activity among multiethnic low-income housing residents. Med Sci Sports Exerc.

[B44] Schneider PL, Crouter SE, Bassett DR (2004). Pedometer measures of free-living physical activity: comparison of 13 models. Med Sci Sports Exerc.

[B45] Emmons KM, Stoddard AM, Fletcher R, Gutheil C, Suarez EG, Lobb R, Weeks J, Bigby JA (2005). Cancer prevention among working class, multiethnic adults: results of the healthy directions-health centers study. Am J Public Health.

[B46] Sorensen G, Barbeau E, Stoddard AM, Hunt MK, Kaphingst K, Wallace L (2005). Promoting behavior change among working-class, multiethnic workers: results of the healthy directions--small business study. Am J Public Health.

[B47] Vernon SW, Meissner H, Klabunde C, Rimer BK, Ahnen DJ, Bastani R, Mandelson MT, Nadel MR, Sheinfeld-Gorin S, Zapka J (2004). Measures for ascertaining use of colorectal cancer screening in behavioral, health services, and epidemiologic research. Cancer Epidemiol Biomarkers Prev.

[B48] Korn EL, Graubard BI (1999). Analysis of Health Surveys.

[B49] Tudor-Locke C, Bassett DR (2004). How many steps/day are enough? Preliminary pedometer indices for public health. Sports Med.

[B50] Emmons KM, Lobb R, Puleo E, Bennett G, Stoffel E, Syngal S (2009). Colorectal cancer screening: prevalence among low-income groups with health insurance. Health Aff (Millwood).

[B51] Shavers VL, Shankar S (2002). Trend in the prevalence of overweight and obesity among urban African American hospital employees and public housing residents. J Natl Med Assoc.

[B52] Lemon S, Zapka J, Puleo E, Luckmann R, Chasan-Taber L (2001). Colorectal cancer screening participation: comparisons with mammography and prostate-specific antigen screening. Am J Public Health.

[B53] Fairfield KM, Fletcher RH (2002). Vitamins for chronic disease prevention in adults: scientific review. JAMA.

[B54] Fletcher RH, Fairfield KM (2002). Vitamins for chronic disease prevention in adults: clinical applications. JAMA.

[B55] Martin AR, Nieto JM, Ruiz JP, Jimenez LE (2008). Overweight and obesity: The role of education, employment and income in Spanish adults. Appetite.

[B56] Sarlio-Lahteenkorva S, Lahelma E (1999). The association of body mass index with social and economic disadvantage in women and men. Int J Epidemiol.

[B57] Bennett GG, McNeill LH, Wolin KY, Duncan DT, Puleo E, Emmons KM (2007). Safe to walk? Neighborhood safety and physical activity among public housing residents. PLoS Med.

[B58] Eyler AA, Baker E, Cromer L, King AC, Brownson RC, Donatelle RJ (1998). Physical activity and minority women: a qualitative study. Health Educ Behav.

[B59] Eyler AA, Matson-Koffman D, Vest JR, Evenson KR, Sanderson B, Thompson JL, Wilbur J, Wilcox S, Young DR (2002). Environmental, policy, and cultural factors related to physical activity in a diverse sample of women: The Women's Cardiovascular Health Network Project--summary and discussion. Women Health.

